# Supplementary material for: EdgeHOG: a method for fine-grained ancestral gene order inference at large scale
Source: Nat Ecol Evol. 2025 Aug 19;9(10):1951–61. doi: 10.1038/s41559-025-02818-0 (PMC12507687; doi:10.1038/s41559-025-02818-0)
Supplement: Supplementary file 1 — Detailed algorithm and benchmarking results. [file 41559_2025_2818_MOESM1_ESM.pdf]

nature ecology & evolution

Supplementary information https://doi.org/10.1038/s41559-025-02818-0

EdgeHOG: a method for fine-grained ancestral gene order inference at large scale

In the format provided by the authors and unedited

Algorithm

Extant synteny graphs

PyHam[1] is called with the orthoXML file of HOGs and the guide species tree (in newick) to model the lineage of all extant genes of all input genomes (**Figure 1**). A lineage is a graph which connects a gene to its parental gene in the upper internal level of the species tree, up until the level of the rootHOG, namely the last common ancestor (LCA) in which the gene is inferred to have emerged. Extant genes not assigned to a HOG are singletons. The genomic coordinates of protein-coding genes in extant genomes are then extracted from either input GFF files or from the HDF5 file of the OMA browser to initialize a linear synteny graph for each leaf of the phylogeny. In such a graph, a node is an extant gene, an edge connects two adjacent genes and each connected component is a chromosome/plasmid/contig (**Figure 2a**).

Ancestral synteny graphs

The bottom-up phase propagates edges of synteny graphs from the leaves to the root of the species tree, creating a synteny network at each level of the species tree. An ancestral synteny network is first initialized as a collection of ancestral genes inferred at the current level of the phylogeny. A copy of the synteny graph of each child is then transformed by removing any gene of degree 2 with no parent and reconnecting its two neighbors with a weight equal to the minimal weight between the removed gene and its previous two neighbors (in case the reconnected edge was already present in the network, this minimal weight is added to the initial edge weight). This operation accounts for the insertion of a gained gene between two ancestral genes. By default, only two consecutive reconnections are allowed, but this can be changed by the user, *e.g.* if one expects events like integrations of a mobile genetic element into the chromosome. Edges connecting genes with a distinct parental gene in each transformed child graph are then propagated to the ancestral synteny network (in case the edge was already propagated from another child, its weight is incremented with the weight of the edge in the current child) (**Figure 2a**). When the --orient_edges option is enabled, the weight of each transcriptional context (co-directional, convergent, and divergent) is propagated along with the absolute, directionless weight. When merging two consecutive edges in cases where a central gene is absent in the parent graph, the resulting edge follows these rules: i) two consecutive co-directional contexts merge into a co-directional context, ii) a divergent context followed by a convergent context (or vice versa) merges into a co-directional context, iii) a divergent context followed by a co-directional context (or vice versa) results in a divergent context, iv) a convergent context followed by a co-directional context (or vice versa) results in a convergent context. If the two edges being merged have non-null weights for multiple contexts, all combinations are considered. In such cases, the resulting edge will retain weights for multiple contexts, with these weights adjusted by a scaling factor. This ensures that their total sum equals the smaller absolute weight of the two original edges.

Parsimonious ancestral synteny graphs

The top-down phase then prunes edges from synteny networks that are not justified by parsimony, from the root to the leaves of the species tree. At any visited level, the algorithm flags edges whose distribution in children levels and/or in the parental level are supportive that the visited level is either the LCA or a descendant of the LCA in which the edge is thought to have emerged. These correspond to edges present in the propagated network of at least two children, or present in the propagated network of one child and whose propagated form is present in the previously pruned network of the parent. In case two adjacent paralogs result from a duplication at the current level, the propagated form of the edge essentially is the single copy parental gene predating the duplication event. Edges not fulfilling the above conditions are removed from the current synteny network (**Figure 2b**). When the --orient_edges option is enabled, the trimming algorithm applies to each edge context. Only parsimoniously supported contexts are retained, with unsupported ones reset to 0. If an edge is supported but none of its contexts are (e.g., convergent in one child, divergent in another), context weights remain unchanged.

Linearized graphs

The linearization phase removes edges from each synteny network until all genes have 2 or less neighbors. First, conflicting genes (degree > 2) are ordered such that easy-to-resolve genes are treated before the hardest cases. This is done by sorting conflicting nodes by decreasing order of node weight (criterion 2), then by increasing order of degree (criterion 1) (**Figure 2c**). It means that nodes with 3 neighbors are resolved before nodes with more neighbors. Neighbors of conflicting nodes are then sorted by increasing order of cumulative edge weights in the linear path passing by the visited neighbor and stopping when a node of degree != 2 is met (criterion 2), then by increasing order of the weight of their edge to the conflicting node (criterion 1). This yields a priority list of edges to remove until the degree of the conflicting node becomes 2 (**Figure 2c**). For example, if a conflicting node has three neighbors—node 'A' with an edge weight of 3, node 'B' with an edge weight of 2 along the path to another node, and terminal node 'C' with an edge weight of 2—the priority list for edge removal would be C, B, and A. Yet, because the notion of best neighbor between two genes may not be reciprocal, this may lead to removal of edges between genes that will end up being singletons or terminal nodes of linear contigs at the end of the linearization process. Therefore, a priority list of removed edges to reintroduce is finally created based on the weight that the linear connected component would have if the removed edge was reintroduced in the graph (criterion 2), and based on the weight of the edge itself (criterion 1). Of note, an edge can be reintroduced in the graph only if the two genes still have less than 2 neighbors after the reintroduction of higher-priority edges from the list. When the --orient_edge option is enabled, the chosen context for the ancestral edge is the one with the highest weight. In case of weight equality, the hierarchy is the following: 1) co-directional, 2) divergent, 3) convergent.

Benchmarking

Validation on simulated genomes

To evaluate the method in a rigorously controlled experiment, we simulated genome evolution resulting in a clade of 100 short extant genomes of ~400 genes with gene losses, gene duplications, translocations and inversions using ALF[2]. We then inferred the gene orders of the 99 ancestral levels of the input guide species tree with edgeHOG and AGORA under default parameters, using HOGs computed from the proteomes of the 100 extant genomes (converted into reconciled gene trees for AGORA). For both tools, we compared the predicted gene order at each internal level of the tree with that of the known order from the simulation (**Figure 3a**). Overall, the harmonic mean precision (percentage of predicted adjacencies being correct) and recall (percentage of real adjacencies being predicted) of edgeHOG reached 98.9% and 96.8%, respectively, while that of AGORA reached 96.0% and 94.9%. In addition, edgeHOG's precision and recall was stable at every depth of the phylogeny, while AGORA's tended to be lower for more recent ancestors (**Figure 3a**). This somewhat counterintuitive behavior was already reported by the authors of AGORA[3]. Indeed, the weight of an ancestral edge in AGORA is not solely influenced by its number of occurrences in descendant leaves, but in all leaves of the species tree as long the edge is found in at least one descendant of the visited internal node. In other words, the closer an ancestral node is to its descendant leaves, the smaller the contribution of its closely related descendants to edge weights, increasing the likelihood of AGORA's linearization process making errors. EdgeHOG, unaffected by this bias, offers an improvement, providing more generalizable results which can be relevant to users interested in reconstructing recent ancestors.

To gain insights in the relative contribution of edgeHOG's three algorithmic phases (bottom-up, top-down, and linearization), we also measured precision and recall after each phase; while recall is already high after the first phase, the analysis makes it clear that the top-down and linearization phases are also needed to achieve the high precision, particularly for deep ancestors (**Figure 3a**).

We also repeated the experiment with a more challenging simulation, increasing the rates of gene duplication, gene loss, translocation, and inversion by an order of magnitude (see following table). On this harder dataset, edgeHOG outperformed AGORA more markedly, achieving a harmonic mean precision of 40.3% and recall of 18.8%, compared to AGORA's 13.9% precision and 3.8% recall (**Extended Data Figure 1**).

Parameters	*“Easy” simulation*	*“Difficult” simulation*
Rate of gene duplication	0.001	0.05
Ratio of translocation after duplication	0.2	0.2
Rate of gene loss	0.005	0.05
Rate of inversion	0.0005	0.01
Rate of translocation	0.0005	0.05

Supplementary Table 6: Change of parameters between the “easy” and the “difficult” simulation

Yeast Gene Order Browser benchmark

To assess edgeHOG on real data, we took advantage of the expert and thorough work performed by the Yeast Gene Order Browser (YGOB) to manually curate the likely gene order in the last common ancestor of a clade of 20 yeast species[4]. We ran edgeHOG and AGORA on the dataset and compared the predicted adjacencies at the root of the species tree with that annotated by the YGOB. Despite the complex evolutionary history of the 20 species, involving an ancient hybridization between two ancestors, followed by whole genome duplication in the last common ancestor of a clade of 12 yeasts[5], the precision and recall of edgeHOG reached 91.7% and 77.5%, respectively, while that of AGORA reached 90.6% and 79.2% (**Figure 3b**). In addition, 99.7% of the ancestral gene adjacencies correctly inferred by EdgeHOG were predicted in the same transcriptional orientation as in the YGOB’s annotation (99.7% for AGORA), showing the high precision of both tools at inferring ancestral gene orientation.

Masked extant gene orders benchmark

Despite the well-established nature of YGOB and its curated ancestral gene order, its focus on a specific yeast clade and evolutionary divergence raises questions about the generalizability of our findings. Additionally, there is a possibility that YGOB might favor the method that has the most in common with its inference process. We thus designed another test on real data encompassing a diverse set of 50 vertebrate genomes and masked the gene order in 10 genomes, *i.e.* treating each gene as if it were on its own contig. We ran edgeHOG and AGORA on this dataset to assess their ability to accurately reconstruct the gene order in these 10 genomes using information from the other 40 genomes. Specifically, we inferred the gene adjacencies of each masked genome by mapping the predicted adjacencies of their most direct ancestor onto the corresponding descendant genes in the masked genome. Under the assumption that an ancestral genome shares a substantial number of gene adjacencies with a direct descendant, all else being equal,

the number of accurate predictions in a masked genome projected from ancestral edges can be considered as a proxy of the quality of the ancestral gene order inference. The 10 genomes were selected to obtain a diverse set of characteristics, in terms of their taxonomic distribution, proteome quality as assessed by OMArk[6], number of sister taxa with respect to the direct ancestor, and degree of polytomy of their direct ancestor (**Figure 4, Extended Data Figure 2**).

Across the 10 masked species, edgeHOG demonstrated slightly higher recall and precision compared to AGORA, with an average increase of +1.5% in precision and +0.4% in recall, (**Figure 3c**). Interestingly, the recall and precision achieved vary significantly across the 10 masked species. As expected, reconstructions become more challenging for species whose most direct ancestor branched deep in the phylogeny. Other factors, such as the polytomy level of the ancestral node, the proteome quality and contiguity level of the masked genome, and the number of terminal duplications, also influence the results (**Extended Data Figure 3**). Of note, the average precision for predicting transcriptional orientations was 98.3% for edgeHOG and 99.0% for AGORA.

We next evaluated how increasing the number of input genomes affects edgeHOG's performance by repeating the 10-species masking experiment using 156 genomes instead of 50 (**Extended Data Figure 2**). Recall improved (+2.1% in average); however, adding more species slightly impacted precision (-0.8% in average) (**Extended Data Figure 4**). Without masking species, we also compared ancestral reconstructions from both datasets and found them largely consistent (average Jaccard index: 0.65, stdev = 0.14), though with some variation across branches. Notably, the 156-species dataset produced more ancestral adjacencies overall, especially for deeper nodes. For instance, edgeHOG inferred 11,051 adjacencies for the last common ancestor of Gnathostomata, versus 8,193 with 50 species (**Extended Data Figure 5**). This demonstrates that comparing more genomes, and thus handling large datasets, has the potential to improve the resolution of ancestral genomes.

References

1. Train, C.-M., Pignatelli, M., Altenhoff, A. & Dessimoz, C. iHam and pyHam: visualizing and processing hierarchical orthologous groups. *Bioinformatics* **35**, 2504–2506 (2019).
2. Dalquen, D. A., Anisimova, M., Gonnet, G. H. & Dessimoz, C. ALF--a simulation framework for genome evolution. *Mol. Biol. Evol.* **29**, 1115–1123 (2012).
3. Muffato, M. *et al.* Reconstruction of hundreds of reference ancestral genomes across the

eukaryotic kingdom. *Nat Ecol Evol* **7**, 355–366 (2023).

4. Byrne, K. P. & Wolfe, K. H. The Yeast Gene Order Browser: combining curated homology and syntenic context reveals gene fate in polyploid species. *Genome Res.* **15**, 1456–1461 (2005).

5. Marcet-Houben, M. & Gabaldón, T. Beyond the Whole-Genome Duplication: Phylogenetic Evidence for an Ancient Interspecies Hybridization in the Baker's Yeast Lineage. *PLoS Biol.* **13**, e1002220 (2015).

6. OMArk, a tool for gene annotation quality control, reveals erroneous gene inference. *Nat. Biotechnol.* (2024) doi:10.1038/s41587-024-02155-w.
