# Supplementary material for: EdgeHOG: a method for fine-grained ancestral gene order inference at large scale
Source: Nat Ecol Evol. 2025 Aug 19;9(10):1951–61. doi: 10.1038/s41559-025-02818-0 (PMC12507687; doi:10.1038/s41559-025-02818-0)
Supplement: Supplementary file 2 — Reporting Summary [file 41559_2025_2818_MOESM2_ESM.pdf]

nature portfolio

Corresponding author(s): Christophe Dessimoz

Last updated by author(s): Jun 11, 2025

Reporting Summary

Nature Portfolio wishes to improve the reproducibility of the work that we publish. This form provides structure for consistency and transparency in reporting. For further information on Nature Portfolio policies, see our Editorial Policies and the Editorial Policy Checklist.

Statistics

For all statistical analyses, confirm that the following items are present in the figure legend, table legend, main text, or Methods section.

n/a	Confirmed	
☐	☒	The exact sample size (*n*) for each experimental group/condition, given as a discrete number and unit of measurement
☐	☒	A statement on whether measurements were taken from distinct samples or whether the same sample was measured repeatedly
☐	☒	The statistical test(s) used AND whether they are one- or two-sided<br>*Only common tests should be described solely by name; describe more complex techniques in the Methods section.*
☒	☐	A description of all covariates tested
☐	☒	A description of any assumptions or corrections, such as tests of normality and adjustment for multiple comparisons
☐	☒	A full description of the statistical parameters including central tendency (e.g. means) or other basic estimates (e.g. regression coefficient) AND variation (e.g. standard deviation) or associated estimates of uncertainty (e.g. confidence intervals)
☐	☒	For null hypothesis testing, the test statistic (e.g. *F*, *t*, *r*) with confidence intervals, effect sizes, degrees of freedom and *P* value noted<br>*Give P values as exact values whenever suitable.*
☒	☐	For Bayesian analysis, information on the choice of priors and Markov chain Monte Carlo settings
☒	☐	For hierarchical and complex designs, identification of the appropriate level for tests and full reporting of outcomes
☒	☐	Estimates of effect sizes (e.g. Cohen's *d*, Pearson's *r*), indicating how they were calculated

Our web collection on statistics for biologists contains articles on many of the points above.

Software and code

Policy information about availability of computer code

Data collection	n/a
Data analysis	In benchmarks, HOGs inference was performed with OMA standalone version 2.6.0 (https://github.com/DessimozLab/OmaStandalone).<br>Ancestral gene order inferences were performed with edgeHOG version 0.1.0 (https://github.com/DessimozLab/edgehog) and AGORA basic workflow version 3.1 (https://github.com/DyogenIBENS/Agora).<br>Functional characterisation of ancestral genes/HOGs (e.g. fraction of descendant genes on organelle contigs, average copy number in extant genomes etc.) was performed using pyHAM version 1.2.0 (https://github.com/DessimozLab/pyham).<br>Gene Ontology Enrichment Analysis (GOEA) in LECA's contigs was performed using goatools version 1.3.1 (https://github.com/tanghaibao/goatools).<br>LECA's contigs were visualized with Cytoscape version 3.10.0 (https://cytoscape.org/).<br>Dating of adjacencies in MiY was performed using the protocole described in https://github.com/DessimozLab/edgehog/blob/main/README.md.<br>All scripts used in the study are available in the Supplementary Dataset 1: https://doi.org/10.6084/m9.figshare.26425081.v2

For manuscripts utilizing custom algorithms or software that are central to the research but not yet described in published literature, software must be made available to editors and reviewers. We strongly encourage code deposition in a community repository (e.g. GitHub). See the Nature Portfolio guidelines for submitting code & software for further information.

Data

Policy information about availability of data

All manuscripts must include a data availability statement. This statement should provide the following information, where applicable:

- Accession codes, unique identifiers, or web links for publicly available datasets
- A description of any restrictions on data availability
- For clinical datasets or third party data, please ensure that the statement adheres to our policy

Simulated ancestral and extant genomes have been generated with ALF (alfsim binary version 4.0, http://alfsim.org), using parameters listed in the Supplementary Dataset 1: https://doi.org/10.6084/m9.figshare.26425081.v2.
The Yeast Gene Order Browser dataset v7-Aug2012 was downloaded from http://ygob.ucd.ie/
All other genomes used in this study are from the OMA database : https://omabrowser.org/oma/home/ (using the OMA Browser database HDF5 file, the species tree in newick and the HOGs orthoxml file available in https://omabrowser.org/oma/archives/All.Nov2022/ and https://omabrowser.org/oma/archives/All.Jul2023/)
Ages in MiY of ancestors were fetched from TimeTree version 5 resource : https://timetree.org
The predicted ancestral gene orders are browsable in https://omabrowser.org/oma/genome/
Data used in analyses are provided in the Supplementary Dataset 1: https://doi.org/10.6084/m9.figshare.26425081.v2

Research involving human participants, their data, or biological material

Policy information about studies with human participants or human data. See also policy information about sex, gender (identity/presentation), and sexual orientation and race, ethnicity and racism.

Reporting on sex and gender	n/a
Reporting on race, ethnicity, or other socially relevant groupings	n/a
Population characteristics	n/a
Recruitment	n/a
Ethics oversight	n/a

Note that full information on the approval of the study protocol must also be provided in the manuscript.

Field-specific reporting

Please select the one below that is the best fit for your research. If you are not sure, read the appropriate sections before making your selection.

☐ Life sciences ☐ Behavioural & social sciences ☒ Ecological, evolutionary & environmental sciences

For a reference copy of the document with all sections, see nature.com/documents/nr-reporting-summary-flat.pdf

Ecological, evolutionary & environmental sciences study design

All studies must disclose on these points even when the disclosure is negative.

Study description	This study presents edgeHOG, a computational tool for fine-grained ancestral order inference at large scale. Utilizing publicly available genomic data, we conducted large-scale analyses which were validated through simulated and empirical data as described in the Methods section. The notion of traditional experimental units or replicates is not applicable to this work
Research sample	2845 genomes from the Jul2023 release of the OMA database (1965 bacteria, 173 archaea, 707 eukaryotes). Validation was performed on smaller datasets as described in the methods section.
Sampling strategy	All available genomes in the OMA database were used to reconstruct ancestral gene orders.
Data collection	n/a
Timing and spatial scale	n/a
Data exclusions	No data was excluded
Reproducibility	Input data, code, and scripts are provided.
Randomization	n/a

Blinding | n/a

Did the study involve field work? ☐ Yes ☒ No

Reporting for specific materials, systems and methods

We require information from authors about some types of materials, experimental systems and methods used in many studies. Here, indicate whether each material, system or method listed is relevant to your study. If you are not sure if a list item applies to your research, read the appropriate section before selecting a response.

Materials & experimental systems

n/a	Involved in the study
☒	☐ Antibodies
☒	☐ Eukaryotic cell lines
☒	☐ Palaeontology and archaeology
☒	☐ Animals and other organisms
☒	☐ Clinical data
☒	☐ Dual use research of concern
☒	☐ Plants

Methods

n/a	Involved in the study
☒	☐ ChIP-seq
☒	☐ Flow cytometry
☒	☐ MRI-based neuroimaging

Plants

Seed stocks | n/a

Novel plant genotypes | n/a

Authentication | n/a
